# Development of a homeolog-specific gene editing system in an evolutionary model for the study of polyploidy in nature

**DOI:** 10.3389/fgeed.2025.1645542

**Published:** 2025-08-29

**Authors:** Shengchen Shan, Michael T. Pisias, Evgeny V. Mavrodiev, Jonathan P. Spoelhof, Bernard A. Hauser, W. Brad Barbazuk, Pamela S. Soltis, Douglas E. Soltis, Bing Yang

**Affiliations:** ^1^ Florida Museum of Natural History, University of Florida, Gainesville, FL, United States; ^2^ Division of Plant Science and Technology, University of Missouri, Columbia, MO, United States; ^3^ Department of Biology, University of Florida, Gainesville, FL, United States; ^4^ Genetics Institute, University of Florida, Gainesville, FL, United States; ^5^ Biodiversity Institute, University of Florida, Gainesville, FL, United States; ^6^ Donald Danforth Plant Science Center, St. Louis, MO, United States

**Keywords:** CRISPR, evolutionary model, genome evolution, homeolog-specific editing, polyploidy, *Tragopogon*

## Abstract

Polyploidy, or whole-genome duplication (WGD), is a significant evolutionary force. Following allopolyploidy, duplicate gene copies (homeologs) have divergent evolutionary trajectories: some genes are preferentially retained in duplicate, while others tend to revert to single-copy status. Examining the effect of homeolog loss (i.e., changes in gene dosage) on associated phenotypes is essential for unraveling the genetic mechanisms underlying polyploid genome evolution. However, homeolog-specific editing has been demonstrated in only a few crop species and remains unexplored beyond agricultural applications. *Tragopogon* (Asteraceae) includes an evolutionary model system for studying the immediate consequences of polyploidy in nature. In this study, we developed a CRISPR-mediated homeolog-specific editing platform in allotetraploid *T. mirus*. Using the *MYB10* and *DFR* genes as examples, we successfully knocked out the targeted homeolog in *T. mirus* (4*x*) without editing the other homeolog (i.e., no off-target events). The editing efficiencies, defined as the percentage of plants with at least one allele of the targeted homeolog modified, were 35.7% and 45.5% for *MYB10* and *DFR*, respectively. Biallelic modification of the targeted homeolog occurred in the T_0_ generation. These results demonstrate the robustness of homeolog-specific editing in polyploid *Tragopogon*, laying the foundation for future studies of genome evolution following WGD in nature.

## 1 Introduction

Polyploidy, also known as whole-genome duplication (WGD), is a major evolutionary force in plants (Soltis et al., 2015; Van de Peer et al., 2021; Morris et al., 2024). WGDs generate genetic, phenotypic, and metabolic diversity and are associated with increased evolvability and diversification (Wendel, 2015; Soltis and Soltis, 2016; Landis et al., 2018; Doyle and Coate, 2019; Fox et al., 2020; Van de Peer et al., 2021; Morris et al., 2024). All living angiosperms are of ancient polyploid ancestry (Jiao et al., 2011), and 35% of extant vascular plant species may have originated via polyploidy (Wood et al., 2009). In addition, most crops are polyploids (Renny-Byfield and Wendel, 2014), and polyploidy plays an important role in plant breeding (Udall and Wendel, 2006; Sattler et al., 2016). Therefore, a better understanding of polyploid genome function and evolution is essential for both comprehending plant diversity and facilitating crop improvement.

In newly formed allopolyploids (those that arose through hybridization of closely related species and associated genome doubling), there are duplicate gene copies (homeologs) with redundant or overlapping expression and function. Over time, these duplicates experience various fates, ranging from retention of both copies with original function to homeolog divergence to loss of one copy via fractionation (Papp et al., 2003; Freeling et al., 2012; Tang et al., 2012; De Smet et al., 2013; Wendel et al., 2018). Some genes are consistently conserved as singletons, implying their single-copy status is advantageous and favored by selection (Paterson et al., 2006; De Smet et al., 2013; Li et al., 2016). In addition, following WGD, genes from one parent may be more highly retained than those from the other (i.e., subgenomic dominance) (Chaudhary et al., 2009; Evangelisti and Conant, 2010; Wendel et al., 2012).

For many other genes, duplicate copies are preserved following WGD, a phenomenon that may be explained by the dosage balance hypothesis, which argues that genes encoding subunits of protein complexes tend to be retained following polyploidy; loss of one copy of these genes may be selected against because of the disrupted stoichiometry of members of multi-subunit protein complexes (Birchler and Veitia, 2007; 2012; 2021). Additionally, genes encoding transcription factors are usually dosage-sensitive and are more likely to be retained in duplicate following WGD: loss of one copy of transcription factor genes may have a pleiotropic effect on many downstream genes involved in the same pathway; this effect would not be observed when losing one copy of genes acting at the termini of genetic networks (Blanc and Wolfe, 2004; Freeling et al., 2012; Birchler and Veitia, 2021).

Despite these observed gene retention patterns, the mechanisms underlying these patterns remain elusive, and the phenotypic consequences of changes in gene dosage are still largely unknown. This is primarily due to: 1) the lack of a homeolog-specific editing system for manipulating gene dosage across polyploid plants, except in a few crop species, including hexaploid wheat (*Triticum aestivum*) (Zhang et al., 2016; Liang et al., 2017) and tetraploid cotton (*Gossypium hirsutum*) (Chen et al., 2025; Yu et al., 2025); and 2) the absence of functional studies in organisms that best exemplify the earliest phases of WGD, especially species from natural systems. A deeper insight into gene fate following recent WGD is critical for understanding the genetic basis of the success of polyploids.

The diploid-polyploid system in North American *Tragopogon* (Asteraceae) represents an evolutionary model for studying the immediate consequences of polyploidy (Ownbey, 1950; Soltis et al., 2004; 2012). The naturally occurring allotetraploids *Tragopogon miscellus* and *T. mirus* formed in the last 95–100 years. The diploid parents of *T. miscellus* are *T. dubius* and *T. pratensis*, and those of *T. mirus* are *T. dubius* and *T. porrifolius*. Previous studies have demonstrated that novel arrays of karyotypes, gene content and expression, and epigenetic regulation were generated in these newly formed *Tragopogon* polyploids (Tate et al., 2009; Buggs et al., 2011; Chester et al., 2012; Spoelhof et al., 2017; Shan et al., 2020; 2024; Yoo et al., 2024). In addition, an efficient CRISPR/Cas9-mediated gene editing system has been recently developed in *T. porrifolius* (2*x*) and *T. mirus* (4*x*), allowing simultaneous editing of both alleles in diploid *Tragopogon* and all four alleles in allotetraploid *Tragopogon* (Shan et al., 2018; Shan et al., 2025).

In this study, we developed a homeolog-specific editing system in allotetraploid *Tragopogon* using *MYB10* and *DFR* (*dihydroflavonol 4-reductase*) genes as examples. Both genes are involved in the well-characterized anthocyanin biosynthesis pathway (Elomaa et al., 2023). This represents the first such system established in a non-model polyploid plant. Our work provides the foundation for future investigations into gene retention patterns following polyploidy, with broad implications for both agricultural applications and evolutionary biology.

## 2 Materials and methods

### 2.1 *Tragopogon* material and seed germination

The allotetraploid *T. mirus* individual used in this study was grown from seed collected from a natural population in Pullman, WA, United States (Soltis and Soltis collection ID: 3091-3). Seed sterilization and germination followed the protocols described in Shan et al. (2018).

### 2.2 Identification of candidate genes in *Tragopogon*

The *DFR* genes in diploid *Tragopogon* species were identified in Shan et al. (2025). The process of identifying *MYB10*, an R2R3 *MYB* transcription factor gene, is described below. All *T. dubius* R2R3 *MYB* genes were identified using the *Gerbera hybrida* (Asteraceae) R2R3 domain (accession no. CAD87010; Elomaa et al., 2023) as the query in a BLASTP search (*e*-value = 1e-10) against the *T. dubius* annotated protein sequences (Spoelhof et al., in prep.). Sequences from *Tragopogon* and 17 additional R2R3 *MYB* gene sequences from model species, including *Solanum lycopersicum*, *Mimulus lewisii*, *G. hybrida*, and *Vitis vinifera* (Lin-wang et al., 2010; Yuan et al., 2014; Naing and Kim, 2018), were aligned using MAFFT (v.7.520; Katoh and Standley, 2013). A maximum likelihood tree was constructed using IQ-TREE (v.2.2.2.7; Minh et al., 2020), and the resulting phylogeny included a clade containing the *Gerbera MYB10* gene and its *T. dubius* orthologs. Six R2R3 *MYB* genes were identified in *T. dubius*. Using the same approach, four R2R3 *MYB* genes were found in *T. porrifolius*. Reciprocal best BLAST hits confirmed orthologous relationships between the *MYB* candidates from *T. dubius* and *T. porrifolius*. All scripts used in this step are available at: https://github.com/GatorShan/EDGE_Project/tree/main/MYB10.

Expression profiles of *T. dubius* and *T. porrifolius* R2R3 *MYB* genes were examined using leaf transcriptomes (BioProject accession: PRJNA210897; Yoo et al., 2024). The trimmed reads from *T. dubius* and *T. porrifolius* were mapped to their respective reference genomes using STAR (v.2.7.11b; Dobin et al., 2013). Mapped reads were quantified using the featureCounts program from the Subread package (v.2.0.6; Liao et al., 2014). Raw counts were then converted to normalized expression values in FPKM (fragments per kilobase of exon per million fragments mapped). In both *T. dubius* and *T. porrifolius*, one R2R3 *MYB* gene exhibited dominant expression in the leaf transcriptomes. This gene was designated as *MYB10* in both diploid species. Additionally, the expression profiles of both *MYB10* homeologs were examined in allotetraploid *T. mirus* using leaf transcriptome data from Yoo et al. (2024): both homeologs were present and expressed in *T. mirus*. All scripts used in this step are available at: https://github.com/GatorShan/EDGE_Project/tree/main/Trag_leaf_transcriptome.

### 2.3 Plasmid construction

Construction of plasmids followed protocols described in Shan et al. (2018). Oligonucleotide sequences used are listed in Supplementary Table S1. For each candidate gene (e.g., *MYB10* and *DFR*), a plasmid was constructed to specifically target the *T. porrifolius* homeolog.

For the plasmid targeting the *T. porrifolius MYB10* homeolog, the cloning process started with two constructs: pCAMBIA1300-Cas9-GFP (destination vector) and pENTR4-AtU6-26 (entry vector). Oligonucleotides gTpoMYB10-F1 and gTpoMYB10-R1 were annealed to generate the double-stranded oligo gTpoMYB10-1. A single SNP between the two homeologs was present at this CRISPR target site. The gTpoMYB10-1 oligo was then integrated into the entry vector, resulting in construct pENTR4-AtU6-26-gTpoMYB10-1.

The sgRNA cassette from the entry vector was then mobilized into the destination vector via a Gateway LR reaction (Thermo Fisher Scientific, Waltham, MA, United States). The final plasmid was named pCAMBIA1300-Cas9-GFP-AtU6-26-gTpoMYB10-1; its sequence was confirmed through whole plasmid sequencing (performed by Plasmidsaurus using Oxford Nanopore Technology). The final construct was introduced into *Agrobacterium tumefaciens* strain EHA105 by electroporation.

For the plasmid targeting the *T. porrifolius DFR* homeolog, the process of plasmid construction was the same except for the use of a different entry vector (pENTR4-AtU6-1-AtU6-29). Oligonucleotides gTpoDFR1-F and gTpoDFR1-R were annealed to form gTpoDFR1; similarly, gTpoDFR2-F and gTpoDFR2-R were annealed to generate gTpoDFR2. The CRISPR target site for gTpoDFR1 contained five SNPs that differed between the two homeologs, while the site for gTpoDFR2 had one SNP. gTpoDFR1 and gTpoDFR2 were then integrated into the entry vector following AtU6-1 and AtU6-29 promoters, respectively. The final entry vector was pENTR4-AtU6-1-gTpoDFR1-AtU6-29-gTpoDFR2. Following the Gateway LR reaction, the final plasmid was named pCAMBIA1300-Cas9-GFP-AtU6-1-gTpoDFR1-AtU6-29-gTpoDFR2.

### 2.4 *Agrobacterium*-mediated transformation and plant regeneration

We followed the methods described in Shan et al. (2025) for transformation and regeneration. Briefly, leaf explants from 4-8-week-old *T. mirus* seedlings were placed on callus induction medium for 2 days. After co-cultivation with *Agrobacterium* (OD_600_ = 0.15–0.20), explants were transferred to co-cultivation medium for 3 days, then moved to selective callus induction medium. After 3 weeks, developing calli were transferred to selective shooting medium to initiate shoot formation. After 2–3 weeks, calli with emerging shoots were moved to shoot elongation medium. Two weeks later, elongated shoots were gradually transferred to selective rooting medium.

All selection and regeneration steps were carried out in a tissue culture incubator under controlled conditions (14 h light/10 h dark, 25 °C; model CU36L4C8, Percival Scientific, Perry, IA, United States). Rooted shoots were then transferred to soil and grown in a growth chamber under the same conditions (model AR-1110, Percival Scientific).

### 2.5 Genotyping regenerated *T. mirus*

To detect the presence of the transgene, a fragment of the transfer DNA (T-DNA) was amplified using genomic DNA extracted from regenerated *T. mirus* plants. For the experiment targeting the *MYB10* gene, primers gTpoMYB10-F1 and GmUbi-R2 were used to amplify a 616-bp DNA fragment of the T-DNA (Supplementary Figure S1A). Each PCR reaction (25 μL) contained 1 μL of genomic DNA (25–125 ng), 1 × Green GoTaq Reaction Buffer (Promega, Madison, WI, United States), 2.5 mM MgCl_2_, 200 μM dNTPs, 0.5 μM of each primer, and 1.25 U Apex Taq DNA polymerase (Genesee Scientific, El Cajon, CA, United States). PCR conditions were as follows: initial denaturation at 95 °C for 3 min; 32 cycles at 95 °C for 30 s, 53 °C annealing for 30 s, and 72 °C extension for 1 min; final extension at 72 °C for 5 min; and hold at 4 °C. Plants were considered transgenic if a band of the expected size (616 bp) was detected by gel electrophoresis. For the experiment targeting the *DFR* gene, primers AtU6-F2 and AtU6-R2 were used to amplify a 476-bp T-DNA fragment (Supplementary Figure S1B). The PCR conditions were the same as above, except the annealing temperature was set at 68.1 °C.

To evaluate editing results for the two candidate genes in allopolyploid *T. mirus*, we genotyped each homeolog separately (Supplementary Figures S2, S3). For the *MYB10* gene, primers TduMYB10-F1 and TduMYB10-R1 were used to amplify the *T. dubius* homeolog (amplicon size: 895 bp) (Supplementary Figure S2A), and primers TpoMYB10-F1 and TpoMYB10-R1 were used to amplify the *T. porrifolius* homeolog (amplicon size: 936 bp) (Supplementary Figure S2B). Each 20-μL PCR reaction contained 1 μL of genomic DNA (20–100 ng), 1 × Phusion HF Buffer (New England Biolabs, Ipswich, MA, United States), 200 μM dNTPs, 0.5 μM of each primer, and 0.4 U Phusion DNA polymerase (New England Biolabs). PCR conditions for both primer sets were as follows: initial denaturation at 98 °C for 30 s; 32 cycles at 98 °C for 10 s, 61 °C annealing for 30 s, and 72 °C extension for 45 s; final extension at 72 °C for 10 min; and hold at 4 °C.

For genotyping the two homeologs of the *DFR* gene in *T. mirus*, primers Tdu-sub_DFR_F1 and Tdu-sub_DFR_R1 were used to amplify the *T. dubius* homeolog (amplicon size: 992 bp) (Supplementary Figure S3A), and Tpo-sub_DFR_F1 and Tpo-sub_DFR_R3 were used for the *T. porrifolius* homeolog (amplicon size: 1,053 bp) (Supplementary Figure S3B). The PCR setting was identical to that used for *MYB10* genotyping, except the annealing temperature was adjusted to 61.5 °C and the extension time was increased to 1 min.

PCR products were sent for Sanger sequencing at Eurofins Genomics, Louisville, KY, United States. Genotypes were inferred by manually inspecting the chromatograms, following the approach described in Shan et al. (2018), with the assistance of the Synthego ICE Analysis tool (v.3; https://ice.editco.bio/#/).

## 3 Results

### 3.1 Homeolog divergence for *MYB10* and *DFR* genes in allotetraploid *Tragopogon mirus*

**FIGURE 1:**
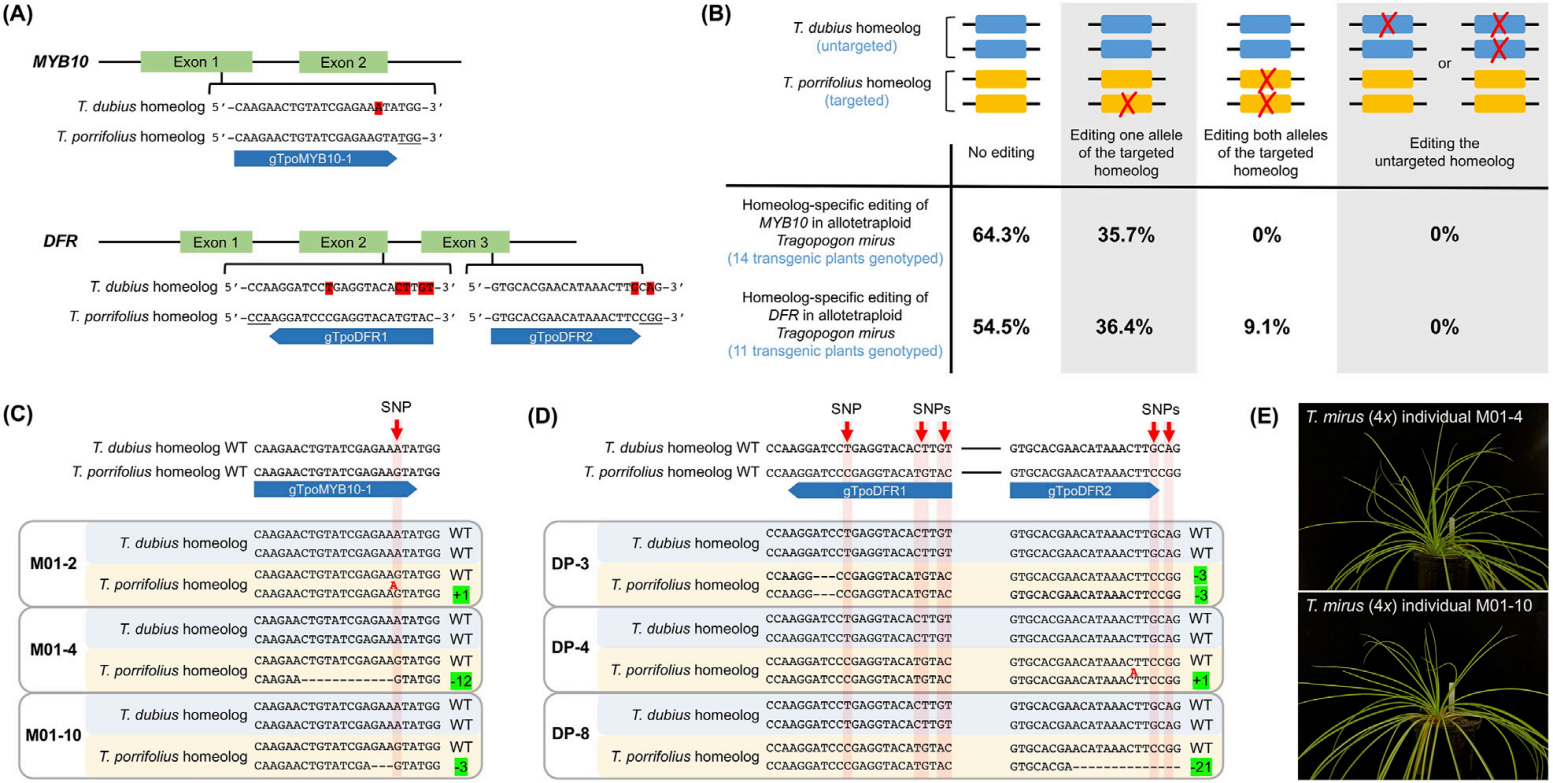
Homeolog-specific editing in allotetraploid *Tragopogon mirus*. **(A)** One CRISPR/Cas9 target site was located within exon 1 of *MYB10*, and the sgRNA gTpoMYB10-1 was designed to specifically target the *T. porrifolius* homeolog in *T. mirus* (4*x*). A single SNP distinguishing the two homeologs at the target site is highlighted in red. For *DFR*, two CRISPR/Cas9 target sites were selected–one in exon 2 and the other in exon 3. Two sgRNAs, gTpoDFR1 and gTpoDFR2, were designed to specifically target the *T. porrifolius* homeolog. SNPs distinguishing the *T. dubius* and *T. porrifolius* homeologs at these target sites are also highlighted in red. PAM sequences are underlined; sgRNAs are represented by blue bars, with the flat end indicating the 5′ end and the arrowhead indicating the 3′ end. **(B)** Genotyping results confirmed homeolog-specific editing of the *MYB10* and *DFR* genes in T_0_ transgenic *T. mirus* (4*x*) plants. Both alleles from the *T. dubius* homeolog are shown as blue rectangles, and those from the *T. porrifolius* homeolog are shown in yellow. Red crosses indicate sequence modifications. Off-target edits (i.e., modification of the untargeted *T. dubius* homeolog) were not detected in either experiment; all editing events occurred in the *T. porrifolius* homeolog. For *MYB10*, 64.3% of transgenic plants showed no edits in either homeolog, while 35.7% had a monoallelic edit in the *T. porrifolius* homeolog. For *DFR*, 54.4% of plants were unedited, 36.4% had monoallelic in the *T. porrifolius* homeolog, and 9.1% exhibited biallelic edits in the *T. porrifolius* homeolog. **(C)** Genotypes of representative *T. mirus* individuals with edits in the *T. porrifolius MYB10* homeolog. The SNP distinguishing wildtype *T. dubius* and *T. porrifolius* homeologs is indicated by the red arrow. Nucleotide insertion is shown in red. **(D)** Genotyping results of representative *T. mirus* individuals with edits in the *T. porrifolius DFR* homeolog. For individual DP-8, not all deleted nucleotides (nt) in the “-21” allele are shown. “-x” denotes an x-nt deletion (e.g., 3-, 12-, or 21-nt), and “+y” indicates a y-nt insertion (e.g., 1-nt). WT: wildtype. **(E)** Photographs of two *T. mirus* (4*x*) individuals (M01-4 and M01-10) with the *T. porrifolius* homeolog of *MYB10* successfully edited.

For *MYB10*, we identified one CRISPR target site in exon 1 (Figure 1A). There is one SNP at this site between the two homeologs in *T. mirus*: A in the *T. dubius* homeolog and G in the *T. porrifolius* homeolog. This SNP is three nucleotides (nt) upstream of the protospacer adjacent motif (PAM) sequence (i.e., TGG), which is present in both homeologs. Utilizing this SNP, we designed single-guide RNA (sgRNA) gTpoMYB10-1 to specifically target the *T. porrifolius* homeolog. Although the CRISPR/Cas9 complex can bind both homeologs (as each contains a PAM site), only the targeted *T. porrifolius* homeolog–where the genomic sequence perfectly matches the spacer sequence of the sgRNA–is expected to be edited.

In terms of the *DFR* gene, two CRISPR target sites from the *T. porrifolius* homeolog were chosen: one is in exon 2, and the other in exon 3 (Figure 1A). We designed a sgRNA, named gTpoDFR1, to specifically target the *T. porrifolius* homeolog in exon 2. At this site, the PAM sequence (TGG) was present on both homeologs. There were five SNPs in the 21-nt protospacer region upstream of the PAM between the two homeologs (Figure 1A). For the second target site in exon 3, CGG PAM was only present in the targeted *T. porrifolius* homeolog. Another sgRNA, named gTpoDFR2, was designed to match the sequence upstream of PAM in the *T. porrifolius* homeolog. One SNP was found between the *T. porrifolius* homeolog and the corresponding region in the *T. dubius* homeolog (Figure 1A).

### 3.2 Homeolog-specific editing of *MYB10* in allotetraploid *T. mirus*

Of the 14 regenerated plants (with both shoot and root) of *T. mirus* on selective media, all were transgenic (Supplementary Figure S1A). Among the 14 plants, five (35.7%) showed edits in the targeted *T. porrifolius* homeolog, while none had edits in the *T. dubius* homeolog (the untargeted copy) (Figures 1B,C). All five genome-edited plants carried a monoallelic mutation, with one of the two alleles from the *T. porrifolius* homeolog modified. The most common mutation was a 12-nt deletion (i.e., −12), observed in three *T. mirus* individuals: M01-4, M01-6, and M01-12 (Figures 1C,E); all three shared the exact same 12-nt deletion. In addition, one mutant *T. mirus* individual (M01-2) had 1-nt insertion (i.e., +1) located three nucleotides upstream of PAM; individual M01-10 had a 3-nt deletion (Figures 1C,E).

### 3.3 Homeolog-specific editing of *DFR* in allotetraploid *T. mirus*

Of the 12 plants of *T. mirus* that we genotyped, 11 were transgenic (Supplementary Figure S1B). Among the 11 plants, five (45.4%) had edits in the targeted *T. porrifolius* homeolog, while none showed modifications in the untargeted *T. dubius* homeolog (Figure 1B). One *T. mirus* individual (DP-3) contained biallelic mutation of the *T. porrifolius* homeolog: 3-nt deletions were found in both alleles at the site targeted by sgRNA gTpoDFR1 (Figure 1D). In addition, four plants had monoallelic mutations at the site targeted by gTpoDFR2: three individuals (DP-4, DP-5, and DP-7) shared the same 1-nt insertion, and one plant (DP-8) had a 21-nt deletion (Figure 1D).

## 4 Discussion

Although CRISPR/Cas-mediated gene editing has been applied in many allopolyploid plants (e.g., Braatz et al., 2017; Sánchez-Gómez et al., 2023; Xu et al., 2024; Zhang et al., 2025), these studies knocked out all copies of the target gene without differentiating between individual homeologs. Homeolog-specific editing has only been reported in a few polyploid crop species, including hexaploid wheat (*T. aestivum*) and allotetraploid cotton (*G. hirsutum*), to study function of genes associated with agronomic traits (Liang et al., 2017; Chen et al., 2025; Yu et al., 2025). *GW2* controls grain weight in wheat. Liang et al. (2017) designed a sgRNA that perfectly matched the *GW2-B1* and *GW2-D1* homeologs but had a single nucleotide mismatch at the target site in the *GW2-A1* homeolog. Following biolistic delivery of the CRISPR/Cas9 ribonucleoprotein complexes, 0%, 2.2%, and 4.4% of the T_0_ wheat plants showed editing events at the A, B, and D homeologs, respectively (Liang et al., 2017). By leveraging SNPs between the two *GhMML3* homeologs, sgRNAs were designed to specifically knock out either the A-subgenome copy (*GhMML3_A12*) or the D-subgenome copy (*GhMML3_D12*) in *G. hirsutum* (4*x*) (Chen et al., 2025). This study demonstrated that *GhMML3_A12* and *GhMML3_D12* regulate fiber development in a dosage-dependent manner. Yu et al. (2025) used CRISPR to edit either the *GhHD1A* or the *GhHD1D* homeolog in *G. hirsutum* and found functional divergence between the two homeologs in regulating trichome and fiber initiation.

Beyond plant systems, CRISPR-mediated allele-specific editing in diploid species (Yoshimi et al., 2014; Smith et al., 2015; Hashizume et al., 2025) and homeolog-specific editing in polyploid species (Gan et al., 2021; 2023) have been reported in several animal models. In an allele-specific editing experiment in the diploid laboratory rat (*Rattus norvegicus*), sgRNAs were designed to target either the *Tyr* ^*C*^ or *Tyr* ^*c*^ allele by exploiting one SNP at the CRISPR/Cas9 target site. Editing was found only in the targeted allele, with editing efficiencies of 30.4% and 28.6% for the *Tyr* ^*C*^ and *Tyr* ^*c*^ alleles, respectively (Yoshimi et al., 2014). Smith et al. (2015) showed that CRISPR/Cas9 can distinguish between two alleles in human cells based on a single nucleotide difference. In polyploid carp (*Carassius gibelio*), sgRNAs were designed to target either the *CgRunx2b-A* or *CgRunx2b-B* homeolog to study their respective roles in intermuscular bone development (Gan et al., 2023).

Currently, homeolog-specific editing studies in polyploid species employ several approaches to differentiate between homeologs. First, the non-PAM approach takes advantage of presence/absence variation in the PAM sequence–only the homeolog containing the PAM site will be edited. In the current study, we used this approach to differentiate the two *DFR* homeologs at the target site in exon 3 (Figure 1A). This method has also been applied in the study of cotton by Chen et al. (2025). The second approach aims to maximize mismatches within the 10 nucleotides immediately upstream of PAM (also known as the seed sequence) in the sgRNA, as targeting specificity is largely determined by this region. Studies in tetraploid cotton (Chen et al., 2025) and hexaploid wheat (Zhang et al., 2016) have employed this strategy. In *T. mirus*, a single SNP within the seed sequence successfully differentiated the *T. dubius* and *T. porrifolius MYB10* homeologs (Figures 1A,B). Finally, the use of premixed CRISPR ribonucleoprotein (RNP) has been shown to improve homeolog editing specificity compared to expression of the CRISPR DNA construct. In wheat, Liang et al. (2017) reported that expression of the CRISPR/Cas9 DNA construct led to a 3.8% editing rate in the untargeted homeolog, whereas the RNP approach showed no off-target edits.

Despite the novelty of applying homeolog-specific gene editing in allotetraploid cotton (*G. hirsutum*), neither Chen et al. (2025) nor Yu et al. (2025) reported homeolog editing efficiency or whether any off-target events (i.e., modification of the untargeted homeolog) were detected. In both cases, homozygous mutant lines were often obtained in subsequent generations, implying monoallelic mutation (i.e., editing one of two alleles of the target homeolog) may have been present in the T_0_ generation (Chen et al., 2025; Yu et al., 2025). In model crop species such as cotton, substantial resource investment has enabled the development of streamlined and highly efficient gene editing platforms, where reporting metrics such as editing efficiency and off-target rate may be considered less critical. However, for researchers working on non-model polyploid plants, including those of evolutionary significance, such information is essential for guiding future genome editing efforts.

In the present study, we demonstrated the feasibility of performing homeolog-specific editing in a non-model plant polyploid system. In allotetraploid *Tragopogon mirus*, the homeolog-specific editing efficiencies were 35.7% and 45.5% for *MYB10* and *DFR* genes, respectively (Figure 1). Biallelic mutation of the target homeolog was detected in the T_0_ generation, and importantly, no transgenic plant showed editing of the untargeted homeolog. This detailed report of homeolog-specific editing in *T. mirus* (4*x*) provides a valuable reference for researchers interested in applying similar technologies to their own species of interest.

One limitation of the current work is the relatively low percentage of biallelic mutants in the T_0_ generation: 0% and 9.1% for *MYB10* and *DFR*, respectively (Figure 1B). In diploid *T. porrifolius* (2*x*), the efficiency of creating *DFR* null mutants–where both alleles are edited–was 100% (Shan et al., 2025). Moreover, when using sgRNAs targeting conserved sequences shared by the *T. dubius* and *T. porrifolius* homeologs, 71.4% of the transgenic *T. mirus* showed edits in all four alleles (from both homeologs) (Shan et al., 2025). Therefore, the relatively low efficiency of homeolog-specific editing was unexpected.

Homeolog-specific editing efficiency in polyploid *Tragopogon* can be improved through several strategies. First, incorporating additional sgRNAs may enhance editing efficiency. Our study showed that, compared to using one sgRNA (targeting *MYB10*), using two sgRNAs (targeting *DFR*) increased the editing efficiency from 35.7% to 45.5% (Figure 1B). Future constructs could include three or four sgRNAs to further increase the frequency of biallelic mutants. Second, while only CRISPR/Cas9 was used here, combining CRISPR/Cas9 and CRISPR/Cas12a systems could broaden targeting options: Cas12a recognizes a TTTN PAM sequence, in contrast to the NGG PAM required by Cas9 (Zhang et al., 2023). Additionally, Cas12a crRNA arrays are more compact than Cas9 sgRNA arrays, allowing the incorporation of more guide RNAs in the construct (Port et al., 2020). Third, monoallelic mutants could serve as explants for a second round of transformation, increasing the likelihood of obtaining biallelic mutants. Finally, additional biallelic mutants are expected in the T_1_ generation, as 25% of progeny from a self-fertilized monoallelic T_0_ plant should carry biallelic mutations.

The availability of homeolog-specific editing in the evolutionary model *T. mirus* (4*x*) now enables us to address key questions in polyploid genome evolution and fractionation. Although the current work does not include a systematic phenotypic analysis of *T. mirus* mutants with modified *DFR* or *MYB10* homeologs, such analyses will be performed in our future work. Several important questions will be addressed, including those listed here. For genes in which retention of both homeologs is predicted to be favored by selection (such as genes encoding subunits of protein complexes or those that are dosage-sensitive), what are the effects of losing one *versus* the other homeolog on co-expression network and phenotype? Does the parental copy matter in terms of resulting network and phenotypic response? Additionally, for genes for which reversion to singleton status is predicted to be favored by selection, both homeologs may still be present and expressed in the early stages of polyploidy, including 95–100-year-old *T*. *mirus*. How do the phenotypes and transcriptomes differ between plants retaining both homeologs and genome-edited individuals with only one homeolog? Finally, in cases where one parental homeolog is preferentially retained or expressed in polyploids, what are the consequences of retaining the non-preferred copy? In summary, homeolog-specific editing opens new avenues for investigating polyploid genome evolution, with significant implications for both basic and applied plant science.

## Data Availability

mRNA sequences of *MYB10* from *T. dubius* (*TduMYB10*) and *T. porrifolius* (*TpoMYB10*) are deposited in GenBank (accession numbers PV761044 and PV761045, respectively); sequencing results of *MYB10* and *DFR* from *T. mirus* mutants are deposited in Dryad (https://doi.org/10.5061/dryad.0vt4b8h9t).
