# Supplementary material for: Development of a homeolog-specific gene editing system in an evolutionary model for the study of polyploidy in nature
Source: Front Genome Ed. 2025 Aug 29;7:1645542. doi: 10.3389/fgeed.2025.1645542 (PMC12426060; doi:10.3389/fgeed.2025.1645542)
Supplement: Supplementary file 1 [file Supplementaryfile1.docx]

Supplementary Material

**Supplementary Figure S1.** Transgene detection in *Tragopogon mirus* (4*x*). PCR followed by gel electrophoresis was employed to detect the transgene in regenerated *T. mirus* individuals. **(A)** Primers gTpoMYB10-F1 and GmUbi-R2 were used to amplify a 616-bp DNA fragment of the transfer DNA. Control 1 (negative control) used the genomic DNA from a wildtype *T. mirus* individual (D13-3) as the template. **(B)** Primers AtU6-F2 and AtU6-R2 were used to amplify a 476-bp DNA fragment of the transfer DNA. Control 1 (negative control) used genomic DNA from a wildtype *T. mirus* individual (D13-5) as the template; control 2 (positive control) used the plasmid DNA from pCAMBIA1300-Cas9-GFP-AtU6-1-gTpoDFR1-AtU6-29-gTpoDFR2 as the template; control 3 (negative control) contained no DNA template.

**Supplementary Figure S2.** *Tragopogon mirus MYB10* homeolog amplification using homeolog-specific primers. **(A)** TduMYB10-F1 and TduMYB10-R1 were used to specifically amplify a fragment of 895 bp from the *T. dubius* homeolog in all *T. mirus* individuals. When *T. dubius* (3040-6-3) genomic DNA was used as the template, the amplicon was present; however, it was absent when *T. porrifolius* (3078) genomic DNA was used as the template. **(B)** Primers TpoMYB10-F1 and TpoMYB10-R1 specifically amplified a fragment of 936 bp from the *T. porrifolius* homeolog in all *T. mirus* individuals. The amplicon was present when *T. porrifolius* (3078) DNA was used as the template but was absent when *T. dubius* (3040-6-3) DNA was used as the template. Negative control (i.e., “N.C.”) contained no DNA template.

**Supplementary Figure S3.** *Tragopogon mirus DFR* homeolog amplification using homeolog-specific primers. **(A)** Tdu-sub_DFR_F1 and Tdu-sub_DFR_R1 were used to specifically amplify a fragment of 992 bp from the *T. dubius* homeolog in all *T. mirus* individuals. When *T. dubius* (3040-6-3) genomic DNA was used as the template, the amplicon was present; however, it was absent when *T. porrifolius* (3078) genomic DNA was used as the template. **(B)** Primers Tpo-sub_DFR-F1 and Tpo-sub_DFR-R3 specifically amplified a fragment of 1,053 bp from the *T. porrifolius* homeolog in all *T. mirus* individuals. The amplicon was present when *T. porrifolius* (3078) DNA was used as the template but was absent when *T. dubius* (3040-6-3) DNA was used as the template. Negative control (i.e., “N.C.”) contained no DNA template.

**Supplementary Table S1.** Sequences of primers used in this study.

| Primer | Sequence |
| --- | --- |
| gTpoDFR1-F | 5'-gattGTACATGTACCTCGGGATCCT-3' |
| gTpoDFR1-R | 5'-aaacAGGATCCCGAGGTACATGTAC-3' |
| gTpoDFR2-F | 5'-gattGTGCACGAACATAAACTTC-3' |
| gTpoDFR2-R | 5'-aaacGAAGTTTATGTTCGTGCAC-3' |
| gTpoMYB10-F1 | 5'-gattCAAGAACTGTATCGAGAAGTA-3' |
| gTpoMYB10-R1 | 5'-aaacTACTTCTCGATACAGTTCTTG-3' |
| Tdu-sub_DFR_F1 | 5'-ACAGGGGGTTGAGATACGA-3' |
| Tdu-sub_DFR_R1 | 5'-CGATAAAAACCCGAAAGATG-3' |
| Tpo-sub_DFR_F1 | 5'-TTTCATCCACCCAAAATAGTGAT-3' |
| Tpo-sub_DFR_R3 | 5'-AGTCATCCTTCACTTTTCTACCG-3' |
| TduMYB10-F1 | 5'-ATTTGAACACGTGCTTCTTTTTC-3' |
| TduMYB10-R1 | 5'-TACTCCTCAATCTTGTCGTTCAA-3' |
| TpoMYB10-F1 | 5'-TTCGAAAACGTGCTTCATTTCA-3' |
| TpoMYB10-R1 | 5'-GGTCAATTTCCTTTTCACGG-3' |
